# IL-23R and IL-17A polymorphisms correlate with susceptibility of ankylosing spondylitis in a Southwest Chinese population

**DOI:** 10.18632/oncotarget.20319

**Published:** 2017-08-18

**Authors:** Bin Yang, Yuanwei Xu, Xinle Liu, Zhuochun Huang, Lanlan Wang

**Affiliations:** ^1^ Department of Lab Medicine, West China Hospital, Sichuan University, 610041 Chengdu, China; ^2^ West China Medical School, Sichuan University, 610041 Chengdu, China

**Keywords:** interleukin-23R, interleukin-17A, ankylosing spondylitis, genetic polymorphisms, C-reactive protein

## Abstract

The association between the IL-23R and IL-17A polymorphisms and ankylosing spondylitis (AS) in the Southwest Chinese Population is still unclear. The purpose of this study is to detect the association between IL-23R and IL-17A polymorphisms and AS. A case–control study consisting of 486 AS patients and 480 healthy controls was performed. We used the high-resolution melting methods (HRM) to genotype five selected single nucleotide polymorphisms (SNPs), rs6693831, rs7517847, rs1884444, rs10889677 in the IL-23R gene and rs2275913 in the IL-17A gene. Meanwhile, the laboratory indexes were recorded. In this study, patients with genotype CC (*p* = 8.574E-8) and allele C (*p* = 3.206E-31) on SNP rs6693831 (IL-23R) showed decreased risk of AS. The genotype TT (*p* = 4.551E-6) and allele T (*p* = 0.02) on SNP rs1884444 (IL-23R) showed significant lower risk of AS. Individuals carrying the allele A of rs2275913 showed higher morbidity of AS (*p* = 0.04). We first detected that rs6693831 and rs1884444 in IL-23R gene and rs2275913 in IL-17A gene have genetic association with AS.

## INTRODUCTION

Ankylosing spondylitis (AS), the most common form of spondyloarthropathy, is an insidiously progressive multi-system inflammatory disorder. AS typically causes back pain, lesion of the sacroiliac joints and axial skeleton, the most common lesion is inflammation on fibrous or fibro-cartilaginous structures [1]. The enthesitis can cause small erosions in the cortical bone, which may lead to over bone reabsorption. In the later stages, osteoproliferation leads to the ossification of ligaments, tendons and joint capsules, and eventually to ankyloses [2, 3].

Though the etiology and pathogenesis of AS remains ambiguous, it is strongly genetically determined [4]. Besides the best known risk factor HLA-B27 (Human Leukocyte Antigen B27), the non-major histocompatibility-complex susceptibility loci such as endoplasmic reticulum aminopeptidase 1 (ERAP1) and IL-23R (interleukin 23 receptor) have been confirmed to contribute to the total genetic risk [5–7]. Furthermore, with the success of genome-wide association studies (GWAS) in recent years, many single nucleotide polymorphisms (SNPs) are identified with genetic contributions to the disease [8].

IL-17A is among the family of IL-17 cytokines and mainly produced by Th17 cells. IL-17 is a pro-inflammatory cytokine which can facilitate the production of IL-6, IL-8, TNF, chemokines, matrix metalloproteinases, and receptor activator of nuclear factorκB ligand [6]. IL-17A has been demonstrated to play a critical role in the progression and pathogenesis of inflammatory diseases [9]. IL-17 is also a critical target for the treatment of ankylosing enthesitis and psoriasis-like dermatitis in mice model [10]. In addition, elevated IL-17 levels were found in the serum of AS patients [11].The gene single-nucleotide polymorphisms (SNPs) may have effect on the expression or function of IL-17A.

IL-23 is a key regulating factor in CD4 T-cell differentiation into the TH17 lymphocyte subsets and can stimulate the activated/effector CD4+ T cells to produce IL-17A [12]. This is further exemplified by the absence of ThIL-17cells in IL-23-deficient p19–/– and p40–/– mice [13]. The IL-23/IL-23R-mediated TH17 pathway could mediate inflammation and play a potential role in the development of AS [11, 13–15]. The recent study showed that the levels of IL-23 and IL-17 in the serum of patients with active AS were elevated [16]. And in subclinical gut inflammation in AS, infiltrating monocytic cells appear to be responsible for the increased IL-23 production [17]. The IL-23 and IL-17 are currently being considered as promising therapeutic targets of AS [18]. The therapeutic strategies based on neutralization of IL-17 or IL-23, have shown encouraging results for treatment of psoriasis, multiple sclerosis, Crohn’s disease and ankylosing spondylitis [19, 20]. Together, these studies support an emerging picture of abnormal IL-17/IL-23 immune activation in AS patients.

Interleukin 23 receptor (IL-23R) is a type I cytokine receptor. The IL-23R gene is located on chromosome 1p31, and the encoded protein pairs with the β1 subunit of IL-12 (IL-12Rβ1) to form a receptor for IL-23, thus conferring IL-23 responsiveness on cells expressing both subunits [21]. IL-23R variants have also been reported to elevate susceptibility to several autoimmune disease, such as IBD, psoriasis and multiple sclerosis [21–23]. We suspect that the SNPs on gene may affect the expression or function of IL-23R as several SNPs in IL-23R gene have been confirmed to be associated with AS [24–26].

Based on the previous researches, we selected five SNPs, four of which are rs6693831, rs7517847, rs1884444, rs10889677 in the IL-23R gene and one is rs2275913 in the IL-17A gene, according to the MAF in PubMed database and our preliminary experiment.

A recent study illustrated that using a combination of genetic, clinical, and functional analyses could possibly uncover how a single-nucleotide polymorphism (SNP) can influence the disease process [27]. Thus, in addition to selected SNPs we also recorded laboratory indexes, including C reaction protein(CRP), serum complement C3 and serum complement C4 of AS patients at the same time.

In this study, we investigated whether the SNPs (rs6693831, rs7517847, rs1884444, rs10889677 and rs2275913) are correlated with the susceptibility of Ankylosing Spondylitis in a Chinese Han Population, and the relationship between the SNPs and the laboratory characteristics of AS patients.

## RESULTS

### Association analysis between IL-23R variants and AS

In the study, two SNPs (rs6693831, rsa884444) on IL-23R gene showed strong statistical associations with AS. In rs6693831 (C>T), a significant difference of genotype distribution was observed (*P* = 8.574E-8), for genotype (CC vs. CT) the OR (95% CI) = 0.625(0.464–0.842); for genotype (CC vs. TT) the OR (95%CI) = 0.385(0.276–0.538). For allele (C vs. T), we noticed that individuals carrying allele T have higher risk of AS, [*P* = 3.206E–31, OR (95%CI) = 0.313(0.256–0.382)]. Regarding rs1884444 (T>G), the genotype distribution between AS and healthy controls is significantly different (*P* = 4.551E-6), for (TT vs. GT) the OR (95%CI) = 0.623 (0.466-0.834), for (TT vs. GG) the OR (95%CI) = 0.335(0.212-0.531)]. For allele (T vs. G), the difference is *P* = 0.02 and OR (95%CI) = 0.800(0.662–0.965). For the other two SNPs (rs7517847, rs10889677) in IL-23R, no significant difference was observed between AS patients and healthy controls. (Table 1)

**Table 1:** genotype and allele frequency of single nucleotide polymorphisms (SNPs) on IL-23R

SNPs	Genotype/Allele	Cases (*n* = 468)	Controls (*n* = 480)	*P*-value	OR (95% CI)
rs6693831	CC	150 (32.1)	230 (47.9)		1
	CT	169 (36.1)	162 (33.8)	**8.57E-08**	0.625 (0.464–0.842)
	TT	149 (31.8)	88 (18.3)		0.385 (0.276–0.538)
	C	269 (50.1)	622 (64.8)	**3.21E-31**	1
	T	467 (49.9)	338 (35.2)		0.313 (0.256–0.382)
rs7517847	TT	155 (33.1)	154 (32.1)		1
	GT	215 (45.9)	206 (42.9)	0.332	0.964 (0.719–1.294)
	GG	98 (20.9)	120 (25.0)		1.232 (0.870–1.745)
	T	525 (56.1)	514 (53.5)	0.268	1
	G	411 (43.9)	446 (45.6)		1.108 (0.925–1.328)
rs1884444	TT	117 (25.0)	201 (41.9)		1
	GT	225 (48.1)	241 (50.2)	**4.55E-06**	0.623 (0.466–0.834)
	GG	66 (14.1)	38 (7.9)		0.335 (0.212–0.531)
	T	579 (61.9)	643 (67.0)	**0.02**	
	G	357 (38.1)	317 (33.0)		0.800 (0.662–0.965)
rs10889677	AA	259 (55.3)	280 (58.3)		1
	AC	172 (36.6)	161 (33.5)	0.582	0.866 (0.659–1.138)
	CC	37 (8.1)	39 (8.2)		0.975 (0.603–1.576)
	A	690 (73.7)	720 (75.1)	0.528	1
	C	246 (26.3)	240 (24.9)		0.935 (0.761–1.149)

Abbreviation: OR, odds ratio; CI, confidence interval.

Significant *p*-values (< 0.05) are highlighted in bold.

### Association analysis between IL-17A variants and AS

SNP rs2275913 inIL-17A shows a significant difference in allele (G vs. A) [*P* = 0.04, OR (95% CI) = 0.825(0.690–0.987)], but the genotype distribution didn’t show any significant difference. (Table 2)

**Table 2:** genotype and allele frequency of single nucleotide polymorphisms (SNPs) on IL-17A

SNPs	Genotype/Allele	Cases (*n* = 468)	Controls (*n* = 480)	*P*-value	OR (95% CI)
	GG	112 (23.9)	138 (28.8)		1
	GA	241 (51.5)	235 (49.0)	0.233	0.791 (0.582–1.076)
rs2275913	AA	115 (24.6)	107 (22.2)		0.755 (0.525–1.085)
	G	465 (48.4)	511 (53.2)	**0.04**	0.825 (0.690–0.987)
	A	495 (51.6)	449 (46.8)		

Abbreviation: OR, odds ratio; CI, confidence interval.

Significant *p*-values (< 0.05) are highlighted in bold.

### The relationship between laboratory test results and the frequency distribution of genotypes among AS patients

The total 468 AS patients were divided into three groups according to genotypes of each SNP. The CRP levels were compared among the different genotypes. The only significant difference was found in SNP (rs6693831), among which the three genotypes (CC vs. CT vs. TT) shows different CRP levels (*P* = 0.030). The mean CRP level of patients is 10.1 mg/dl (the range 2.9–25.3 mg/dl) higher than 8.00 mg/dL. The high level of CRP prove that the test has been done during the active term of the disease. Patients with genotype CT presented higher CRP levels (Mean = 26.145, SD = 34.394) compared with CC (Mean = 17.950, SD = 30.905) and TT (Mean = 20.969, SD = 31.611) (Table 3).

**Table 3:** CRP level of AS patients with different genotypes of single nucleotide polymorphisms (SNPs) on IL-23R

	rs6693831					rs7517847				rs1884444				rs10889677			
	CC		CT	TT	*P*-value	TT	GT	GG	*P*-value	TT	GT	GG	*P*-value	AA	AC	CC	*P*-value
CRP(mg/L)	17.9 ± 30.9	26.1 ± 34.4		21.0 ± 31.6	0.03	19.4 ± 29.9	21.3 ± 31.2	24.8 ± 36.1	0.467	21.8 ± 32.9	20.2 ± 30.6	24.6 ± 33.2	0.478	19.2 ± 28.2	19.2 ± 28.2	23.9 ± 36.4	0.95

Data are presented as mean ± SD, comparisons between groups were made by the Kruskal-Wallis test as appropriate.

## DISCUSSION

In the study we observed significant association between rs6693831 (IL-23R) and rs1884444 (IL-23R) and rs2275913 (IL-17A) polymorphisms and AS susceptibility. Furthermore, patients with rs6693831 CT genotype on IL-23R present significantly higher levels of CRP compared with patients with CC and TT genotype. The results above indicate that IL-23R and IL-17A correlate with AS susceptibility in a Chinese Han population.

Ankylosing spondylitis (AS) is a polygenic disease. Based on previous studies, IL-23R has been considered as a potential risk factor for AS. Several polymorphisms in IL-23R gene have been reported correlated with the risk of AS (rs11209032, rs11209026, rs7517847, rs2201841 in IL-23R) [25, 26]. We further studied four SNPs in IL-23R and the results are very encouraging. Two additional SNPs (rs6693831C>T and rs1884444T>G) in IL-23R gene showed significant associations with AS (*P* < 10E-6). A research by Kim, E. S., et al. showed the similiar results which GG+GT genotype of IL23R rs1884444 was associated with the development of intestinal BD [28]. There has been some research of the mechanism, the mutation on rs11209026 has been testified to change Arg381 into Gln381, which subsequently interferes the interaction between IL23R and JAK2 and results in a reduction in cellular response to IL-23 [29]. In our study the SNP rs1884444 was located at codon 3 in exon2 of IL23R. The T to G base change of rs1884444 may disrupt an exonic splicing enhancer and result in exon skipping, malformation or transcript alternative splicing [30]. And this polymorphism results in a change of amino acid from His to Gln, which is responsible for the signal peptide of IL-23R, thereby modulating the pro-inflammatory effects of Th17 cell [30]. The IL-23R rs1884444T/G polymorphism has been reported to be associated with various diseases, including cavitary lesion of pulmonary tuberculosis in Chinese Uygurs [31], Alzheimer’s disease in Han Chinese [32], and increased risk of hepatocellular carcinoma [33]. They may share some of the same mechanisms, and further research on the roles of rs1884444 polymorphism in AS may help solve the problem out. To our knowledge, the SNP rs6693831 association with diseases has not been reported. This is the first study to report that SNP rs6693831 is associated with AS and further functional studies are needed.

With regard to IL-17A, there’s no previous study investigating the correlation between IL-17A gene polymorphisms with AS. Our study revealed that allele G on SNP (rs2275913G>A) on IL-17A was significantly associated with AS susceptibility. Previous studies had shown that the percentage of IL-17 positive CD4+T cells in the peripheral blood mononuclear cells PBMCs and concentrations of IL-17 in supernatants are significantly higher in patients with AS compared with healthy controls [34]. Therefore, we propose a hypothesis that the SNP rs2275913 has an impact on the expression of IL-17 and could be a potential therapeutic target for AS.

In our study of laboratory data, the mean CRP level of patients is 10.1 mg/dl, higher than 8.00 mg/dL which proves that the test has been done during the active term of the disease. we found that AS patients with CT genotype on rs6693831 have higher CRP levels (*P* = 0.022), which brings up a hint that this SNP may relate to the expression or regulation of CRP among AS patients. Previous studies have demonstrated that CRP polymorphisms could influence serum CRP levels and the disease activity in AS, and polymorphism on rs3091244 can also affect CRP levels [35]. These facts suggest that there may exist mutual SNPs, which influence both the IL-23 and CRP at the same time. Meanwhile, CRPs level can be biomarker of inflammation and correlate with baseline radiographic damage [36], so the patients with CT genotype on rs6693831 may present higher levels of inflammation. However, there is a limitation that we did not include the clinical manifestations of AS patients into the genotypic associated analysis, so we do not analyze the correlation between genotypes of studied SNPs and clinical indexes of AS patients.

To conclude, in this study, we observed strong associations between SNPs rs6693831C>T, rs1884444T>G, rs2275913G>A and AS susceptibility. Patients of genotype CT on rs6693831 present higher levels of serum CRP. Those discoveries may represent potential pathogenic factors for AS. Further functional studies may help clarify the role of them in the pathogenesis of AS.

## MATERIALS AND METHODS

### Subjects

A total of 468 patients with AS, fulfilling the modified New York Criteria (1984), all patients were first-diagnosed and haven’t received any kind of NASAIDs treatments or immunotherapy. Any patients who had been diagnosed with any other autoimmune diseases are excluded. Among the included patients 98.29% (460/468) are HLA-B27 positive (detected by BD FACSCanto Flow Cytometers). Meanwhile, 480 sex-, age-, ethnicity-matched healthy controls were enrolled in the current study. The healthy controls were excluded if they have any kinds of immunological diseases. Laboratory test data are recorded among AS patients, including CRP, C3 and C4 levels (detected on the Beckman IMMAGE). All study subjects were recruited from West China Hospital of Sichuan University in Chengdu, China from October, 2013 to October, 2014. Demographic data and clinical characteristics of the AS patients and controls are shown in Table 4. The study was approved by the Institutional Ethics Committee of West China Hospital of Sichuan University and complied with the Declaration of Helsinki. Written informed consent was obtained prior to enrollment from all subjects.

**Table 4:** Demographic characteristics of the AS patients and controls

Characteristics	Cases(*n* = 468)	Controls(*n* = 480)	*P* value
Sex (male/female)	320/148	327/153	0.944
Age, years, mean ± SD	32.75 ± 11.18	37.84 ± 14.31	0.692
HLA-B27 (+)	460/468	0/480	**< 0.001**
CRP (mg/L)	10.1 (2.9–25.3)^*^	----	
C3 (g/L)	1.108 ± 0.280	----	
C4 (g/L)	0.255 ± 0.092	----	

Data was analysed by Chi-square tests.

Data are presented as mean ± standard deviation and median (interquartile range)^*^.

### DNA extraction

Blood samples (3 mL) were collected in EDTA-coated tubes, and genomic DNA was isolated from whole blood samples using the whole blood DNA kit (Bioteke; Beijing, China). DNA was extracted from 200 μL of the whole blood according to the manufacturer’s protocol. The extracted DNA was assessed for purity, yield, and concentration on a spectrophotometer (Bio-RAD; Hercules, CA, USA). Purity was monitored by the A260/A280 ratio. DNA was diluted to 10 ng/ml for working solutions, and the isolated DNA was stored at −20°C.

### Polymerase chain reaction and high-resolution melting genotyping

The polymerase chain reaction (PCR) and high-resolution melting assay were performed under the same conditions in a 96-well plate on the Light Cycler 480 (Roche Diagnostics; Penzberg, Bavaria, Germany). The primers for the amplifications were designed using the software Primer Premier 5.0. The specificity of the primers was investigated on Primer-BLAST (http://www.ncbi.nlm.nih.gov/tools/primer-blast). The detailed information of the primers was shown in Supplementary Table 1. Reaction mixtures contained 1.0 μL purified genomic DNA (10 ng/μL), 0.1 μL forward primer, 0.1 μL reverse primer, 5.0 μL Master Mix (contained FastStart Taq DNA polymerase, reaction buffer, dNTP mix and High Resolution Melting Dye), 1.2 μL 25 mM MgCl_2_, and 2.6 μL H_2_O. Real-time PCR was performed with the following conditions: an initial denaturation step at 95°C for 15 min, continued with 50 cycles of 95°C for 10 s, 60°C for 15 s, and 72°C for 20 s. After the amplification phase, a melting curve analysis was performed at 95°C for 1 min, 40°C for 1 min, 65°C for 1 s, and finally slow heating at 0.01°C/s to 95°C. Genotype of the amplicon was analyzed using the Light Cycler 480 Gene Scanning software v1.2 (Roche Diagnostics). The genotype of each subset was defined according to known genotypes of controls.

### Statistical analysis

The differences in allele and genotype frequencies between cases and controls were assessed by Chi-square tests. Odds ratios (ORs) were calculated with 95% confidence intervals (95% CIs). Continuous data were expressed as mean ± standard deviation (SD) or median and interquartile range. Laboratory data were analyzed by one-way ANOVA or by Kruskal—Wallis tests when homogeneity of variance is significantly different. The above analyses were conducted by SPSS 17.0 software. For all experiments, *P* < 0.05 was considered as statistically significant.

## SUPPLEMENTARY MATERIALS TABLE


