# Supplementary material for: IL-23R and IL-17A polymorphisms correlate with susceptibility of ankylosing spondylitis in a Southwest Chinese population
Source: Oncotarget. 2017 Aug 18;8(41):70310–6. doi: 10.18632/oncotarget.20319 (PMC5642556; doi:10.18632/oncotarget.20319)
Supplement: Supplementary file 1 [file oncotarget-08-70310-s001.pdf]

# IL-23R and IL-17A polymorphisms correlate with susceptibility of ankylosing spondylitis in a Southwest Chinese population

## SUPPLEMENTARY MATERIALS

**Supplementary Table 1: Sequences of primers used for systematic search for SNPs in IL-23R and IL-17A**

| SNP | Forward primer (5′→3′) | Reverse primer (5′→3′) |
|---|---|---|
| **rs6693831 (C>T)** | GCGGTAGTTACGGTCACCTTG | GAGAACCACCCTAGCAGTGAAAC |
| **rs7517847 (G>T)** | TTCCCTTCATACCTACCATCTCAC | GAATTTGAGGGGCCTAGGAGAC |
| **rs1884444 (G>T)** | CAACAGTCTTTTCCTGCTTCCA | CGATACATAAAACACCATACCTCAT |
| **rs10889677(C>A)** | GTGACATTTCTGTGCTCCTACCAT | ACCATGAAGCATGTTCCACCTT |

Abbreviations: OR, odds ratio; CI, confidence interval
Significant *p*-values (< 0.05) are highlighted in bold
